# Exploring Global Research Trends in Burnout among Nursing Professionals: A Bibliometric Analysis

**DOI:** 10.3390/healthcare9121680

**Published:** 2021-12-04

**Authors:** Delana Galdino de Oliveira, Augusto da Cunha Reis, Isabela de Melo Franco, Ayala Liberato Braga

**Affiliations:** 1 Faculty of Administration and Accounting Sciences, Federal University of Rio de Janeiro, Rio de Janeiro 22290-240, Brazil; 2 Federal Center for Technological Education Celso Suckow da Fonseca, Production Engineering Departament, Rio de Janeiro 20271-110, Brazil; augusto.reis@cefet-rj.br; 3 Institute of Applied Social Sciences, Federal Rural University of Rio de Janeiro, Seropédica 23897-000, Brazil; ifranco@ufrrj.br

**Keywords:** burnout syndrome, nursing professionals, bibliometric study

## Abstract

Nursing professionals are constantly exposed to several risk factors and high levels of stress that can affect their mental, emotional, and physical health, which can trigger burnout syndrome. This article aims to use bibliometric analysis to investigate burnout research trends among nursing professionals worldwide and to compare the contributions of different countries/institutions, scientific journals, authors, keywords, and citations. A bibliometric study was performed using the Scopus and Web of Science databases, in the period up to November 2021, aiming to search original and review articles in the English language regarding burnout in nursing professionals. The analysis was performed with a sample of 1406 articles. The most cited article indicated that 43% of nurses had high burnout scores, and a similar percentage were dissatisfied with their work. The most productive and most cited country in the world was the United States of America. Regarding the 10 most cited documents, there were no studies that could provide interventions to reduce burnout in nursing professionals, which can result in a need to develop studies on prevention capable of mitigating the problem, in view of the impacts generated on their mental, emotional, and physical health.

## 1. Introduction

Professionals working in the health area, in general, are exposed to high levels of occupational stress [1], especially nursing professionals who perform their activities under a high level of pressure and risk [2]. These professionals need to deal with different demands that can affect their emotional and psychological state and lead to burnout syndrome (BS).

The term burnout, described by Herbert Freudenberger in the 1970s, is a psychological syndrome resulting from a delayed response to chronic interpersonal stressors present in the work environment. This response encompasses some dimensions, which can be characterized by emotional/overwhelming exhaustion, depersonalization, feelings of cynicism, detachment from work, sense of ineffectiveness, and lack of accomplishment [3,4].

In addition, it is important to highlight people who struggle with depression. This group may also experience burnout, and can underestimate the severity of their condition, not seeking medical help when self-identifying as “burned out”. Therefore, the line between burnout and depression must be carefully considered and not be distinguished [5].

Furthermore, BS is included in the eleventh revision of the International Classification of Diseases (ICD-11), being a problem related to employment or unemployment, resulting from chronic workplace stress not successfully managed [6].

Regarding the chronic workplace stress, the prolonged exposure to stressful situations can hurt mental, physical, and emotional health, and may also have a significant impact on the performance of nursing professionals [7,8,9]. Due to the responsibilities, plus the high work demand, nurses are known to have a higher risk of triggering BS when compared to other professionals [10,11].

The increasing shortage of nursing professionals [12] and the intention to leave work [13] became problems for the health system, with the emerging need to build mechanisms so that this workforce can have resilience [14] and job satisfaction. Several studies associate BS with the intention to leave and the increased turnover [15,16,17,18,19]. However, the COVID-19 pandemic considerably raised the demand for nurses to perform their activities with quality [20]. Frontline professionals involved in treating patients with COVID-19 reported increased insomnia and poorer health [21]. The pandemic increased the level of stress and the workload, which could generate high levels of anxiety among the nursing staff [8]. Despite this, there are insufficient information and data on burnout predictors and risk factors during the pandemic [22].

In this context, there is a need to promote quality of life in the work environment, considering that the performance of these professionals is reduced when they go through this exhaustion process, which may increase the probability of committing medical errors [23]. To alleviate this situation, it is essential to identify risk factors and establish mechanisms to prevent occupational stress. Some studies indicate that their possible causes are: excessive workload [24,25]; professional dissatisfaction [26]; insufficient material and human resources [27,28]. In addition, health managers must also take into account that professionals affected with BS generate an increase in costs for maintaining the health service due to leaves, absenteeism, and turnover [29,30].

The determination of risk factors can configure valuable knowledge to deal with psychological discomfort, which, over long periods, can become an obstacle for the health system, and professionals affected with BS may request medical leave or absence, burdening coworkers. Burnout and occupational stress among health professionals are increasingly recognized as significant threats to patient safety [31] and quality of care [32].

Thus, it is essential to develop studies that provide the mapping and analysis of scientific production related to BS among health professionals, especially in emergencies or outbreaks [33]. The analysis of the scientific production of the literature can contribute with data and information on a given topic. Bibliometrics is a vital tool to assess the evolution of a given subject, based on its intellectual and social contributions [34]. The objective of this research is to identify the scientific production on BS among nursing professionals, examining the contributions of different countries/institutions, scientific journals, authors, keywords, citations, and trending topics.

## 2. Materials and Methods

### 2.1. Study Design 

The bibliometric study aimed to map and investigate the scientific production of burnout syndrome in nursing professionals. The bibliometric analysis provided statistics that summarized publications and analyzed the literature [35]. It consisted of a tool to explore information in scientific studies through quantitative approaches [36].

### 2.2. Study Source

The databases selected to carry out the research were Scopus and Web of Science. The choice of these databases was due to the number of indexed journals and because of the broad coverage of international publications relevant to the subject.

### 2.3. Search Strategy

The sample included the articles of the databases up to 9 November 2021, using search terms in English combined through Boolean operators. The databases were used to retrieve the relevant publications using the following search string: (TITLE: “burnout” OR “burn-out” OR “burn out” OR “professional burnout” (MeSH Terms) OR “occupational burnout” (MeSH Terms) OR “burnout, occupational” (MeSH Terms) OR “career burnout” (MeSH Terms) OR “burnout, career” (MeSH Terms)) AND (TITLE: (“nurse” OR “nursing” OR “nurses” (MeSH Terms)). The research query conducted in the titles aimed to minimize the risk of obtaining false-positive results (irrelevant ones) [33].

### 2.4. Data Collect

The study included: (i) original and review articles; (ii) articles in the English language only; (iii) articles that comprehended any aspect of BS in nursing professionals. Exclusion criteria consisted of publications that did not meet the inclusion criteria, as well as duplicated publications. The articles were retrieved from the databases by two researchers to avoid biases in the selection of articles to be evaluated.

Meetings were held for debate and agreement among researchers about the inclusion or exclusion of studies. In case of disagreements that could not be resolved by means of a consensus, a third researcher was called to improve decision-making. After eliminating nonrelevant studies, the dataset for bibliometric analysis was formulated.

**Figure 1:**
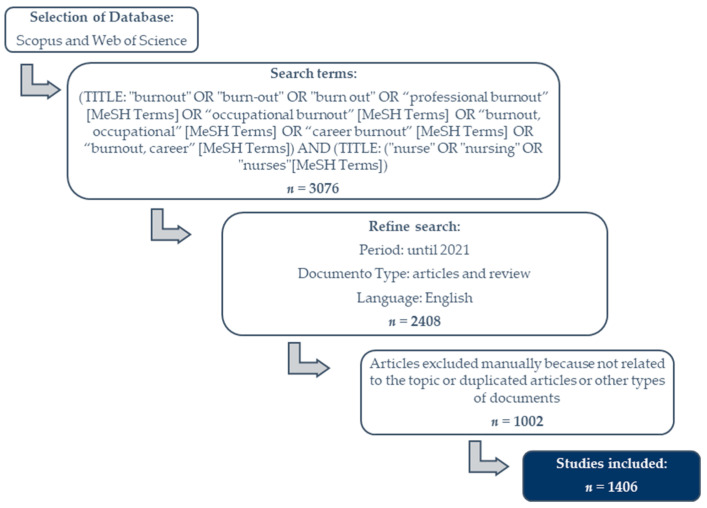
Bibliometric review sampling flowchart.

Figure 1 presents a flowchart with the steps of the process for selecting articles aligned with the research question. In the eligibility process, a total of 3076 articles (1705 on Scopus and 1371 on Web of Science) were identified. After inserting the filters according to the inclusion criteria, 2408 articles (1379 on Scopus and 1029 on Web of Science) were retrieved. A preliminary analysis of the studies was developed based on the reading of titles and abstracts, with 1002 documents being excluded because they were not related to the research topic or they were duplicated articles. At the end of the steps, this bibliometric review included 1406 studies to develop the analysis, as seen in the following flowchart.

### 2.5. Bibliometric Analysis

Data were collected and exported to software called bibliometrix R-package. The package offered a set of tools that allowed the generation of descriptive analysis, statistical graphs, and science mapping, along with the development of research and quantitative analysis in bibliometrics [36].

Bibliometrics is a type of analysis based on identifying information in the literature about a particular area [37,38]. Bibliometric methods are used to quantitatively evaluate scientific production [39]. It generates analysis, including statistical methods that use specific indicators to obtain information about research activity [40]. It allows the discovery of emerging trends in articles and journals, making it possible to explore knowledge in a given area from the analysis of existing literature [41].

This analysis can identify the most productive authors, institutions, countries, and journals in an area of knowledge, can assess the impact of journals, can identify citation patterns, and can identify research topics and research trends based on literature. [42]. In recent years, this method has drawn considerable attention from nursing researchers [43].

## 3. Results

Our study selected the following scientific indicators to analyze the documents: volume and types of publication; annual scientific production; top 10 most relevant sources; top 10 source impact; top 10 most global and local cited publications; authors’ production over time; scientific production by country; top 10 most relevant institutions; most cited country; mapping of the 10 most frequent keywords of the authors and databases; and trending topics.

### 3.1. Main Information about Data

The search query found 1406 publications. The most recurrent document type was article (*n* = 1313; 93%) and review (*n* = 93; 7%). These documents showed a total of 46,066 references and were published in 580 sources (journals, books, etc.). The average citation per document was 32.07 in the period from 1978 to 2021, and they were written by 4343 authors, with a mean of 0.324 documents per author, according to Table 1.

### 3.2. Annual Scientific Production

**Figure 2:**
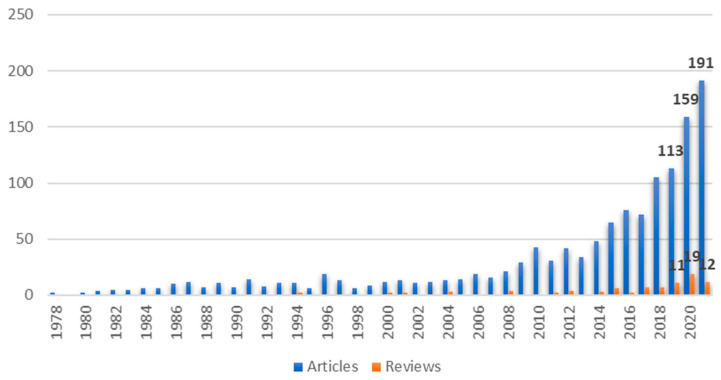
Annual scientific production.

Figure 2 shows the number of articles published per year until 2021. The first publications in the databases took place in 1978. In 2021, 203 studies (191 articles and 12 re-views) were published, corresponding to the largest production in the period analyzed. The annual growth rate was 11.63%, indicating an exponential growth rate, especially in the last 3 years, with 124 papers published in 2019, 178 papers published in 2020, and 203 papers published in 2021. The number of publications per year remained below 50 publications until 2013, when there was a considerable increase in the number of publications. In 2021, the number of publications was more than double in comparison to 2017, and it was more than quadruple compared to 2012. The first review was published in 1988. The years 2020 and 2021 received the highest number of reviews: 19 and 12, respectively.

### 3.3. Top 10 Most Relevant Sources

**Figure 3:**
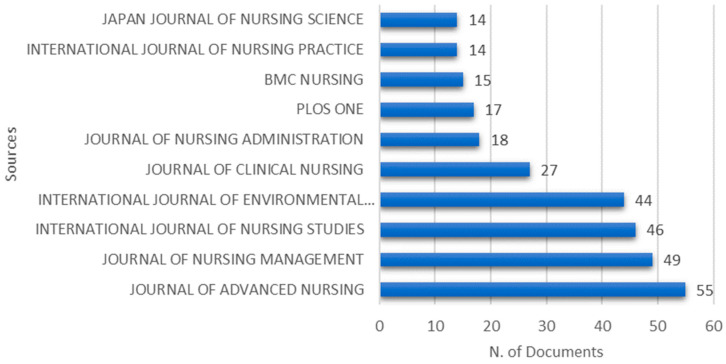
Top 10 most relevant sources.

The retrieved published papers gathered 580 different sources. Figure 3 shows the top 10 most relevant sources, that is, the ones that most contributed with published documents. The number of publications that the 10 leading journals produced was 299, representing 21.27% of the total number of publications by 2021. The three most productive sources were the “Journal of Advanced Nursing”, with 55 articles published in the period of 1978–2021, followed by the “Journal of Nursing Management” (*n* = 49; 3%) and the “International Journal of Nursing Studies” (*n* = 46; 3%). Those three journals are within the subject area of nursing.

### 3.4. Top 10 Source Impact

The retrieved publications received 45,085 citations. The average number of citations per document was 32.07. Table 2 presents the top 10 source impact, based on the number of citations. As shown in the table, the three most cited sources were “Journal of Advanced Nursing” (*n* = 4185; 9%), “International Journal of Nursing Studies” (*n* = 4011; 9%), and “Journal of The American Medical Association” (*n* = 3178; 7%), beginning citations in 1985, 1987, and 2002, respectively.

### 3.5. Top 10 Most Global and Local Cited Publications

This study investigated global and local citation of publications. The global citation refers to the number of citations an article received considering the entire database. On the other hand, local citation measures the number of citations a paper received considering the data included on the survey [44].

Table 3 shows the top 10 most global and local cited publications. It is important to mention that the ranking was made based on the local citations. The three most globally cited articles received 3178, 603, and 435 citations, respectively. The three most local cited publications obtained 127, 86, and 85 citations, respectively. The ranking of the two most cited articles is the same, both in the local and global citations.

The first most cited article was “Hospital Nurse Staffing and Patient Mortality, Nurse Burnout, and Job Dissatisfaction” published in the Journal of the American Medical Association in 2002. The second most cited article was “Nurse burnout and patient satisfaction” published in 2004 in the Medical Care. The third most cited publication was “Nurse turnover: The mediating role of burnout” published in the International Journal of Nursing Studies in 2009.

Among the 10 most cited papers analyzed, it was possible to identify studies about the association between different variables and factors related to nurse retention [45]. Another study found that the mediation model of burnout was able to predict nurse turnover intentions [47]. There were studies aiming to identify the effect of work environment on nurses’ burnout and its impact on patient satisfaction [46]; to assess burnout levels among nurses, as well as related variables [48]; and to explore the relationship between exhaustion of nurses and the quality of care [49].

Besides that, other studies had the objective of verifying the prevalence of burnout in emergency nurses and identifying its specific determinants [10]; of identifying the determinants of BS in nurses acting in the Intensive Care Unit (ICU) [50]; of comparing burnout with general and specific aspects related to job satisfaction, in addition to evaluating levels of burnout and job satisfaction [51]; and of evaluating the applicability of the Maslach Burnout Inventory (MBI) in nursing studies [52]

Lastly, one study revealed that certain explanatory selected variables were responsible for 41.9% of emotional exhaustion, 16.4% of depersonalization, and 25.6% of personal accomplishment in the study sample randomly selected, consisting of 510 psychiatric nurses [53].

### 3.6. Authors’ Production over Time

**Figure 4:**
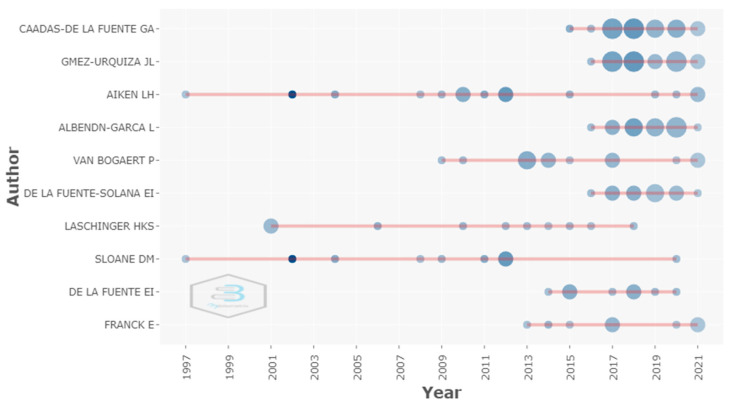
Top authors’ production over time.

Figure 4 shows the production of the main authors over time. The size or diameter of the circle represents the number of articles, and the dark blue color identifies the most cited article per year. The first circle in the line means that, when the author started to publish on the analyzed topic, the larger the circle, the greater the number of papers published per year by author.

It also presents the results of the 10 most productive researchers in studies on burnout among nurses from 1978 to 2021. Authors Aiken and Sloane contributed in 2002 to the article entitled “Hospital Nurse Staffing and Patient Mortality, Nurse Burnout, and Job Dissatisfaction”, receiving the highest number of citations. Therefore, the shade of blue is darker. The result revealed that, since 1997, the authors have contributed with publications on the subject. Most researchers have contributed to the body of research in this field mainly in the last five years. In 2018 and 2017, Cañadas-De La Fuente and Gomez-Urquiza produced a total of four documents in each year and received the third highest citation count per year: 67.25. and 46.8, respectively.

### 3.7. Scientific Production by Country

**Figure 5:**
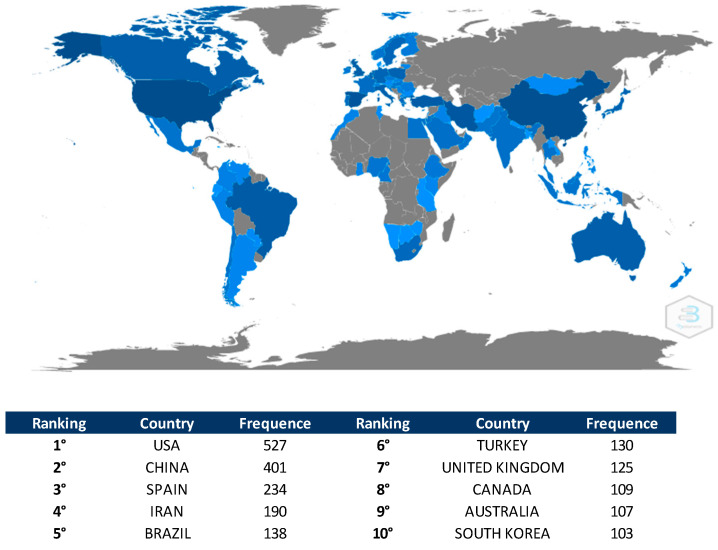
Scientific production by country.

Eighty-one countries—according to all author affiliations—contributed to the development of research on the subject. These countries are geographically distributed in North America, South America, Asia, Europe, Africa, and Oceania. Figure 5 shows the scientific production by country. The map was built through “Biblioshiny”, a web interface of the bibliometrix package. Several shades of blue can indicate different productivity rates. Therefore, the darker the shade of blue, the greater the productivity. On the other hand, the gray color represents that there are no articles. The three most productive countries were the United States of America (USA) (*n* = 527), China (*n* = 401), and Spain (*n* = 234).

### 3.8. Top 10 Most Relevant Institutions

**Figure 6:**
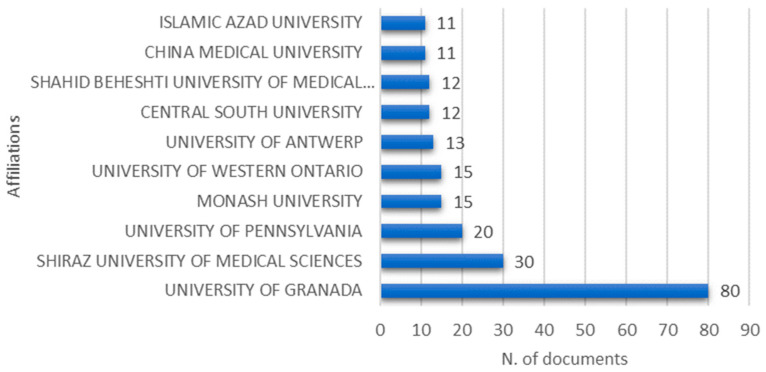
Top 10 most relevant institutions.

Figure 6 lists the top 10 most relevant institutions. University of Granada was the most relevant institution (*n* = 80; 6.0%), followed by Shiraz University of Medical Sciences (*n* = 30; 2.0%) and University of Pennsylvania Central (*n* = 20; 1.0%). Three of the top ten most relevant institutions were in Iran, two in China, one in the USA, one in Australia, one in Belgium, one in Spain, and one in Canada.

### 3.9. Most Cited Country

**Figure 7:**
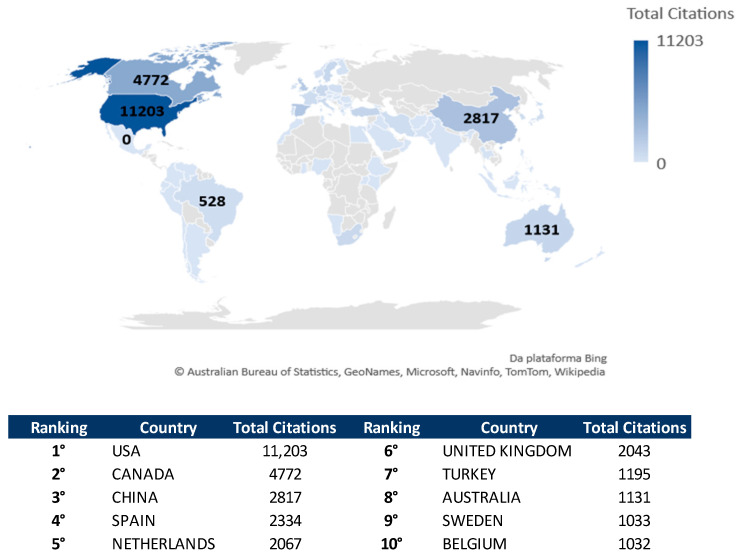
Most cited countries.

Figure 7 shows the top 10 most cited countries that contributed to the development of research on the topic. The three most cited countries were the United States of America (*n* = 11,203 citations), Canada (*n* = 4772 citations), and China (*n* = 2817 citations). The number of citations in the USA was more than double that in Canada, and nearly four times the total citations from China.

### 3.10. Mapping of the 10 Most Frequent Authors’ Keywords and Keywords Plus

Table 4 shows the mapping of the 1hn0 most frequent keywords from authors and databases (keywords plus). The most common keyword used by the authors was “burnout” (*n* = 795), followed by “nurses” (*n* = 284). On the other hand, the keywords provided by the databases with greater frequency were “burnout” (*n* = 1772), followed by “female” (*n* = 1203). The selection of keywords aimed to reflect the focus of the entire study performed and can be identified as future research trends [54]. Therefore, the keywords pro-vided bring an idea of the aspects covered in the studies, such as stress, compassion fatigue, job satisfaction, and job burnout, among others.

### 3.11. Trending Topics

**Figure 8:**
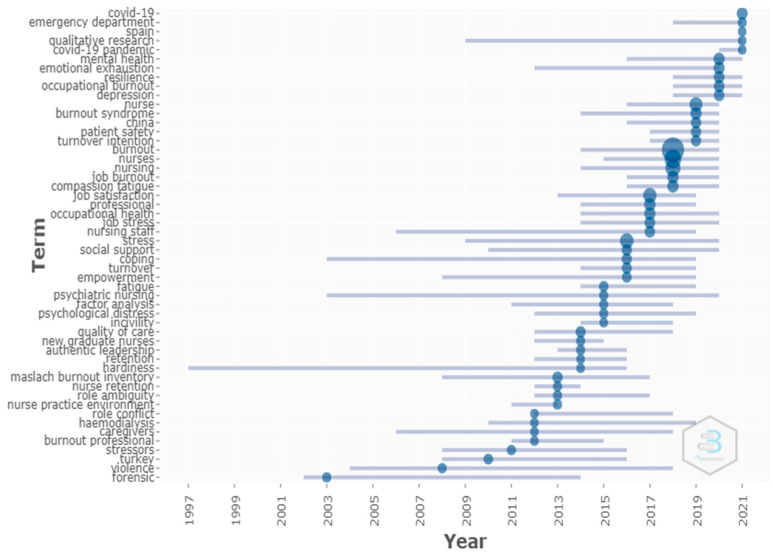
Trending topics.

Figure 8 shows the trending topics. The size or diameter of the circle represents the frequency of the terms provided by the authors in the keywords, following the Biblioshiny parameters of five words minimum frequency and five words per year. In 2019, terms such as nurse (*n* = 85), burnout syndrome (*n* = 39), China (*n* = 19), patient safety (*n* = 18), and turnover intention (*n* = 17) were observed in the surveys. In 2020, it was possible to identify the following terms: mental health (*n* = 34), emotional exhaustion (*n* = 32), resilience (*n* = 27), occupational burnout (*n* = 25), and depression (*n* = 22). In 2021, the highlighted terms were COVID-19 (*n* = 30), emergency department (*n* = 6), Spain (*n* = 5), qualitative research (*n* = 5), and COVID-19 pandemic (*n* = 5). In March 2020, the World Health Organization (WHO) declared the COVID-19 pandemic [55]. The studies produced may reflect the impacts of this situation, considering that many health professionals work directly with patients affected by the disease. Recent studies indicate that, while facing the pandemic, some health professionals developed fatigue, exhaustion [56], and psychological distress [57,58]. The lack of knowledge about how the pandemic can impact on the mental state of health professionals is a research gap [59].

## 4. Discussion

Initially, 3076 documents were counted in the databases using the search terms. After verifying the articles obtained based on the inclusion and exclusion criteria, 1406 papers were used to perform the bibliometric analysis. Among the publications retrieved for this study, 93% were articles and 7% were review.

Concerning the research developed in the sample, some considerations can be observed and extracted. The first articles were published in 1978. The annual scientific production increased over the analyzed time (1978–2021), indicating an exponential growth rate (11.63%). Therefore, it is clear that there is a concern to produce knowledge on the subject, considering that the BS can influence the mental health of the professional, the work performed, and the safety of the patient cared for in different health institutions.

In order of importance, the five most productive countries were as follows: USA, China, Spain, Iran, and Brazil. The five most cited included USA, Canada, China, Spain, and Netherlands. In the bibliometric indicator of the ten most relevant institutions, three institutions were located in Iran, two in China, one in the USA, one in Australia, one in Belgium, one in Spain, and one in Canada. Although Spain appeared with only one institution, it was the most productive affiliation, contributing with 72 articles.

The results showed no standardization for the selection of keywords provided by both authors and databases, that is, words with the same meaning. This is the case, for in-stance, for the keywords provided by the authors: “burnout” and “burnout syndrome”. The “keywords plus” provided by the database showed studies about female and male professionals.

The 10 most relevant sources contributed with 21.26% of the total publications by 2021. The 10 most cited sources contributed with 41.55% of the total citations. Only six journals appeared both among the 10 most cited and among the 10 most relevant: The Journal of Advanced Nursing, the Journal of Nursing Management, the International Journal of Nursing Studies, the International Journal of Environmental Research and Public Health, the Journal of Clinical Nursing, and the Journal of Nursing Administration.

The publications received a total of 45,085 citations, an average of 3207 citations per article. The most cited article was published in the Journal of the American Medical Association. This journal received the third highest number of citations. However, this source did not appear among the 10 most relevant ones, that is, when analyzed by the number of documents published.

The article that obtained the highest number of citations in both sample (local) and databases (global) was published in 2002 [45]. Among the results of this study, it is note-worthy that nurses working in hospitals with higher patient-to-nurse rates are more than twice as likely to suffer from work-related burnout and almost twice as likely to be dissatisfied with their jobs compared to nurses working in hospitals that have lower rates. In addition, the study indicated that 43% of the nurses had high burnout scores and a simi-lar percentage were dissatisfied with their work.

Another study pointed out that 32.8% of the nursing staff members who work in the Intensive Care Unit trigger severe BS. The ICU is an extremely stressful place and may contribute with a high rate of BS to the team [50]. Burnout syndrome is a complex problem and an important predictor in modern workplaces, substantially increasing its prevalence in the recent years [47,52], especially among nursing professionals [48].

Prevalence was measured in a study performed with nurses from six countries: USA, Canada, United Kingdom (Scotland and England), Germany, New Zealand, and Japan. Japan had the highest burnout levels in nurses. On the other hand, the lowest burnout levels were with the German nurses. Nurses from Canada, the United Kingdom, and New Zealand had lower burnout levels than nurses from Japan and the USA, but higher burnout than the German ones [49].

It was highlighted that BS is multi-factorially determined [53]. With this in mind, some risk factors can trigger BS, such as work overload, value conflicts, injustice, lack of support, and inadequate rewards [10,47]. In this sense, the role of the nurse team managers is crucial to keep the work environment less hostile [60,61].

The concern with the worsening of nurse shortages and the intention to leave was mentioned in some studies [45,46,47]. Some changes in the work environment can reduce professional burnout and the intention to leave, as well as produce positive effects on patient satisfaction [46]. When nurses are exhausted and dissatisfied with working conditions, patient satisfaction becomes much lower [51].

From 2019 onwards, it was possible to observe the trend of studies addressing terms in keywords, such as occupational burnout, patient safety, intention to leave, COVID-19, emergency room, resilience, emotional exhaustion, mental health, and depression. It was noteworthy that these terms demonstrate what could be developed in the study and may indicate future research trends. In the case of the term COVID-19, there is likely an increase in the number of publications evaluating the impact of this pandemic on the professionals’ mental health.

Some studies compared the frequency of BS between physicians and nurses [62,63,64]. Other studies indicated the prevalence of BS among nurses [20,65,66,67,68,69]. Aspects such as resilience [70], depression [71], trauma [72], and the role of leadership [73] were also observed in the articles. Finally, studies on factors associated to BS [27,74] and risk factors were found [75].

After a month working in the frontlines of the COVID-19 pandemic, some nurses experienced moderate to severe emotional exhaustion and depersonalization. [76]. During the outbreak, the levels of anxiety and depression presented by health professionals were significantly higher [77]. However, it becomes a challenge to offer support or interventions that can contribute to diminishing stress and exhaustion, since there is a lack of time due to high work demands [78].

There are some limitations in the current study; for instance, the research included only articles in English in the sample. Even though English is a language mostly used in international databases, there could certainly be articles in other languages with more contributions. Besides that, only the Scopus and Web of Science databases were used to carry out the study. It would be interesting to develop this analysis on other databases to verify if the obtained results are similar.

## 5. Conclusions

This study analyzes the research and scientific production trends on burnout syndrome among nursing professionals. Based on bibliometric indicators, publications were investigated at a global level indexed in the Scopus and Web of Science databases. This research indicates that, although the first article was published in 1978, research on BS had a substantial growth from 2007 onwards.

Most of the retrieved studies are inclined to the prevalence, predictors, associated factors, and determinants. In the sample, there are few investigations that provide strategies for prevention, which indicates the importance of developing specific studies on interventions in nursing teams, especially the ones trying to reduce stress and burnout in these professionals.

Currently, COVID-19 appears as the most used term in research, demonstrating the possibility of developing studies that associate burnout with the pandemic. The analyzed sample displays studies that evaluate nursing professionals during the pandemic. More-over, there were few studies during the outbreak addressing the topic.

For future research, it is interesting to use the methodology in other databases, thus making the results broader and disseminating what has been produced, thereby enabling the increase in the evaluated sample. There is also the possibility of identifying other aspects and analyses that were not considered in this study.

## Figures and Tables

**Table 1:** Main information about the bibliometric study.

Description	Results
MAIN INFORMATION ABOUT DATA	
Timespan	1978–2021
Sources (journals, books, etc.)	580
Documents	1406
Average years from publication	8.45
Average citations per documents	32.07
Average citations per year per doc	3656
References	46,066
DOCUMENT TYPES	
Article	1313
Review	93
DOCUMENT CONTENTS	
Keywords Plus (ID)	2249
Authors Keywords (DE)	1699
AUTHORS	
Authors	4343
Author appearances	5402
Authors of single-authored documents	154
Authors of multi-authored documents	4189
AUTHOR COLLABORATION	
Single-authored documents	170
Documents per author	0.324
Authors per document	3.09
Coauthors per document	3.84
Collaboration Index	3.39

**Table 2:** Top 10 source impact.

Journal	Total Citations	PY_Start ¹
Journal of Advanced Nursing	4185	1985
International Journal of Nursing Studies	4011	1987
Journal of The American Medical Association	3178	2002
Journal of Nursing Management	1961	2000
Journal of Nursing Administration	1448	1981
Journal of Clinical Nursing	1215	2002
Medical Care	818	2004
International Journal of Environmental Research and Public Health	795	2013
Health Affairs	587	2011
American Journal of Critical Care	536	2004

¹ PY_start = year that started.

**Table 3:** Top 10 most global and local cited publications.

Ranking	Article Title	Authors	Year	Local Citations	Global Citations	LC/GC Ratio (%)
1	Hospital Nurse Staffing and Patient Mortality, Nurse Burnout, and Job Dissatisfaction	Aiken; Clarke; Sloane; Sochalski; Silber [45]	2002	127	3178	4.00
2	Nurse burnout and patient satisfaction	Vahey; Aiken; Sloane; Clarke; Vargas [46]	2004	86	603	14.26
3	Nurse turnover: the mediating role of burnout	Leiter; Maslach [47]	2009	85	385	22.08
4	Risk factors and prevalence of burnout syndrome in the nursing profession	Cañadas-De la Fuente; Vargas; Concepción San Luis; Inmaculada García; Cañadas; De la Fuente [48]	2015	77	211	36.49
5	Nurse burnout and quality of care: cross-national investigation in six countries	Poghosyan; Clarke; Finlayson; Aiken [49]	2010	58	294	19.73
6	Determinants and prevalence of burnout in emergency nurses: a systematic review of 25 years of research	Adriaenssens; Gucht; Maes [10]	2015	57	338	16.86
7	Burnout Syndrome in Critical Care Nursing Staff	Poncet; Toullic; Kentish-Barnes; Timsit; Pochard; Chevret; Schlemmer; Azoulay [50]	2007	56	435	12.87
8	Nurses’ Widespread Job Dissatisfaction, Burnout, And Frustration With Health Benefits Signal Problems For Patient Care	McHugh; Kutney-Lee; Cimiotti; Sloane; Aiken [51]	2011	45	379	12
9	Factor structure of the Maslach burnout inventory:An analysis of data from large scale cross-sectional surveys of nurses from eight countries	Poghosyan; Aiken; Sloane [52]	2009	44	212	20.75
10	Burnout in psychiatric nursing	Kilfedder; Power; Wells [53]	2001	43	170	25.29

**Table 4:** Mapping of the 10 most frequent authors’ keywords and keywords plus.

Ranking	Authors’ Keywords	Frequency	Ranking	Keywords Plus	Frequency
1°	burnout	795	1°	burnout	1772
2°	nurses	284	2°	female	1112
3°	nursing	183	3°	adult	1109
4°	stress	110	4°	human	989
5°	job satisfaction	102	5°	male	971
6°	nurse	85	6°	nursing staff	807
7°	professional	42	7°	professional	732
8°	job burnout	41	8°	humans	673
9°	burnout syndrome	39	9°	article	623
10°	compassion fatigue	39	10°	middle aged	592

## Data Availability

The data presented in this study are available on request from the corresponding author.
